# Clinical implication of plasma exchange on life-threatening antineutrophil cytoplasmic antibody-associated vasculitis

**DOI:** 10.1186/s12890-020-01181-z

**Published:** 2020-05-28

**Authors:** Pil Gyu Park, Byung-Woo Yoo, Jason Jungsik Song, Yong-Beom Park, Sang-Won Lee

**Affiliations:** 1 Department of Internal Medicine, Yonsei University College of Medicine, Seoul, Republic of Korea; 2 Division of Rheumatology, Department of Internal Medicine, Yonsei University College of Medicine, Seoul, Republic of Korea; 3 Institute for Immunology and Immunological Diseases, Yonsei University College of Medicine, Seoul, Republic of Korea

**Keywords:** Antineutrophil cytoplasmic antibody, Vasculitis, Plasma exchange, Diffuse alveolar haemorrhage

## Abstract

**Background:**

We assessed the rate of and predictors for all-cause mortality in patients with antineutrophil cytoplasmic antibody (ANCA)-associated vasculitis (AAV) receiving plasma exchange (PLEX) and evaluated the survival benefit of PLEX for diffuse alveolar haemorrhage (DAH) between AAV patients receiving PLEX and those not receiving PLEX.

**Methods:**

We retrospectively reviewed the medical records of 212 patients with AAV. Demographic, clinical and laboratory data at the time of PLEX were collected from nine patients receiving PLEX, six of whom had DAH. The follow-up duration was defined as the period from the time of PLEX or DAH occurrence to death for the deceased patients and to the last visit for the survived patients.

**Results:**

The median age of nine AAV patients receiving PLEX was 71.0 years, and five patients were men. Four of nine patients receiving PLEX died at a median follow-up duration of 92.0 days. Three patients died of sepsis and one died owing to a lack of response to PLEX. When patients with DAH receiving or not receiving PLEX were compared, there were no significant differences in variables between the two groups. The cumulative patients’ survival rate between patients with DAH receiving and not receiving PLEX were also compared using the Kaplan-Meier survival analysis; however, no survival-benefit of PLEX for DAH was observed.

**Conclusion:**

The rate of all-cause mortality in nine AAV patients receiving PLEX was found to be 44.4% and the notion that PLEX is beneficial for the improvement in the prognosis of AAV-related DAH was deemed controversial.

## Background

Antineutrophil cytoplasmic antibody (ANCA)-associated vasculitis (AAV) is one of the small vessel vasculitides and comprises microscopic polyangiitis (MPA), granulomatosis with polyangiitis (GPA) and eosinophilic granulomatosis with polyangiitis (EGPA) [1, 2]. The latest recommendations for the management of AAV were proposed by the European League Against Rheumatism (EULAR) and the European Renal Association-European Dialysis and Transplant Association (ERA-EDTA) in 2016 (the 2016 EULAR/ERA-EDTA recommendations) [3]. According to these recommendations, a combination of glucocorticoid with either cyclophosphamide (CYC) or rituximab (RTX) should be administered to patients newly diagnosed with life-threatening AAV. Once remission is achieved, azathioprine, methotrexate or RTX could be maintained and glucocorticoid may be tapered. In particular, in cases of renal failure with rapidly progressive glomerulonephritis (RPGN) or diffuse alveolar haemorrhage (DAH), plasma exchange (PLEX) should be considered [3]. In addition to the 2016 EULAR/ERA-EDTA recommendations, the 2012 Kidney Disease: Improving Global Outcomes (KDIGO) clinical practice guideline for glomerulonephritis recommends PLEX for patients with rapid and severe renal vasculitis and suspected anti-glomerular basement membrane (anti-GBM) glomerulonephritis [4]. Therefore, PLEX is not usually prescribed but is strongly recommended for patients with life-threatening AAV.

With regard to RPGN, the Methylprednisolone versus Plasma Exchange (MEPEX) clinical trial compared the efficacy of PLEX and methylprednisolone pulse therapy in 137 AAV patients with serum creatinine > 5.8 mg/dL and reported that PLEX for induction exhibited a higher frequency of renal recovery [5]. In addition, another clinical trial reported that PLEX had a preventive efficacy against the exacerbation of renal dysfunction in AAV patients with serum creatinine < 5.7 mg/dL in GPA patients [6]. In contrast, a long-term observational clinical study, PLEX was found to have no significant benefit in AAV patients with serum creatinine > 5.7 mg/dL or on dialysis [7]. Moreover, recently, the data from the Plasma Exchange and Glucocorticoids for Treatment of Anti-Neutrophil Cytoplasm Antibody (ANCA)-Associated Vasculitis (PEXIVAS) clinical trial reported that PLEX had no efficacy for reducing the risk of both end-stage renal disease or death in 704 AAV patients [8]. With regard to DAH, several case series reported the positive efficacy of PLEX on DAH [9, 10]. In particular, a retrospective study including 20 AAV patients with DAH reported that combination therapy with PLEX and immunosuppressive drugs was beneficial for lung outcomes [11]. In contrast, a retrospective cohort study including 53 AAV patients with severe alveolar haemorrhage reported no significant difference in mortality between the PLEX and non-PLEX groups [12]. Therefore, the efficacy of PLEX in life-threatening AAV remains controversial.

To date, there has been no study investigating the efficacy of PLEX on life-threatening AAV, such as RPGN and DAH, on a large number of patients, except for a few case reports from Korea. Hence, in this study, we investigated two clinical implications of PLEX on life-threatening AAV. First, we assessed the rate of and predictors for all-cause mortality in AAV patients receiving PLEX. Second, we assessed the survival-benefit of PLEX for DAH between AAV patients receiving PLEX and those not receiving PLEX.

## Methods

### Patients

We reviewed the electronic medical records of 212 AAV patients from the retrospective Severance Hospital ANCA-associated VasculitidEs cohort and selected nine AAV patients who had received PLEX for life-threatening AAV-related clinical symptoms based on the following inclusion and exclusion criteria: i) patients who had been first classified as having AAV at the Division of Rheumatology, Department of Internal Medicine, Yonsei University College of Medicine, Severance Hospital, from October 2000 to March 2019; ii) patients who fulfilled the 2007 European Medicines Agency algorithm for AAV and polyarteritis nodosa (the 2007 EMA algorithm) and the 2012 revised International Chapel Hill Consensus Conference vasculitides definitions [1, 13]; iii) patients who had well-documented medical records with which clinical and laboratory data at diagnosis could be reviewed and Birmingham vasculitis activity score (BVAS) version 3 and five-factor score (FFS) at the time of diagnosis could be calculated [14, 15]; iv) patients who had the results of tests for myeloperoxidase (MPO)-ANCA and proteinase 3 (PR3)-ANCA [16]. We also selected four AAV patients, who had exhibited DAH but not received PLEX, and compared their clinical and laboratory data with those of six AAV patients, who had exhibited DAH and received PLEX. This study was approved by the Institutional Review Board of Severance Hospital (4–2017-0673), who waived the need for patient written informed consent, as this was a retrospective study.

### Clinical and laboratory data

Age and gender were recorded as demographic data at PLEX. Variant type, ANCAs, BVAS, FFS and each clinical manifestation at PLEX were assessed. Reasons for PLEX were also investigated. Methylprednisolone pulse therapy and the administration of immunosuppressive drugs before and at or after PLEX were evaluated. All immunosuppressive drugs that had been administered before the initiation of PLEX were included in ‘Administered immunosuppressive drugs before PLEX’ regardless of induction or maintenance therapy. The follow-up duration was defined as the period from the time of PLEX for DAH, RPGN and cardiac tamponade to death for the deceased patients and as that to the last visit for the survived patients. For AAV patients with DAH not receiving PLEX, the follow-up duration was defined as the period from the time of detection of DAH to death for the deceased patients and to the last visit for the survived patients. The rate and aetiology of all-cause mortality were assessed.

### Statistical analyses

All statistical analyses were conducted using SPSS (version 23 for Windows; IBM Corp., Armonk, NY, USA). Continuous variables were expressed as a median (interquartile range (IQR)), and categorical variables were expressed as the number and percentage. The univariable Cox hazards model analysis was performed to appropriately obtain the hazard ratios (HRs) of each variable for all-cause mortality. Significant differences in categorical variables between the two groups were analysed using the Chi-square and Fisher’s exact tests. Significant differences in continuous variables between the two groups were analysed using the Mann-Whitney test. A comparison of the cumulative patients’ survival rate between the two groups was performed using the Kaplan-Meier survival analysis.

## Results

### Baseline characteristics

Clinical and laboratory data are described in Table 1. Nine of 212 AAV patients (4.3%) received PLEX for life-threatening AAV. The median age was 71.0 years, and five patients were men. Six patients were classified as having MPA and three patients were done as GPA but none as EGPA. ANCAs were detected in six patients; however, anti-GBM was not found. The median BVAS and FFS at PLEX were 20.0 and 3.0, respectively. FFP was used in all patients and there were no PLEX-related complications including bleeding. Five patients received PLEX nine times, two patients received it six times and two patients received it three times. Six patients received PLEX for DAH, two for RPGN, and one for cardiac tamponade. Methylprednisolone pulse therapy was performed at PLEX for all nine patients. The most commonly administered immunosuppressive drugs before and at or after PLEX were CYC and RTX respectively. Four of nine patients receiving PLEX died at a median follow-up duration of 92.0 days. Three patients died of sepsis and one died owing to a lack of response to PLEX. Three patients deceased patients with DAH were all admitted to the intensive care unit (ICU) and received ventilation care along with continuous renal replacement therapy in ICU.

**Table 1:** Characteristics of 9 patients with AAV at the time of PLEX

Variables	Values
**Demographic data at PLEX**	
Age (years)	71.0 (25.0)
Male gender (N, (%))	5 (55.6)
**AAV variants (N, (%))**	
MPA	6 (66.7)
GPA	3 (33.3)
EGPA	0 (0)
**Gap-time from diagnosis to PLEX (days)**	69.0 (127.0)
**ANCA and anti-GBM within 4 weeks before PLEX (N, (%))**	
MPO-ANCA	4 (44.4)
PR3-ANCA	2 (22.2)
ANCA negativity	3 (33.3)
Anti-GBM	0 (0)
**AAV related indices at PLEX**	
BVAS	20.0 (14.0)
FFS	3.0 (1.5)
**Clinical manifestations at PLEX (N, (%))**	
General	9 (100)
Cutaneous	1 (11.1)
Mucous membrane/Eyes	0 (0)
ENT	3 (33.3)
Pulmonary	7 (77.8)
Cardiovascular	3 (33.3)
Abdominal	0 (0)
Renal	7 (77.8)
Nervous systemic	2 (22.2)
**Reason for PLEX (N, (%))**	
DAH	6 (66.6)
RPGN	2 (22.2)
Pericarditis with cardiac tamponade	1 (11.1)
**Methylprednisolone pulse therapy at PLEX (N, (%))**	9 (100)
**Administered immunosuppressive drugs before PLEX (N, (%))**	
CYC	4 (44.4)
RTX	3 (33.3)
AZA	1 (11.1)
MMF	1 (11.1)
TAC	1 (11.1)
None	4 (44.4)
**Administered immunosuppressant drugs at or after PLEX (N, (%))**	
CYC	1 (11.1)
RTX	5 (55.6)
AZA	1 (11.1)
MMF	1 (11.1)
TAC	0 (0)
None	2 (22.2)
**Follow-up duration (days)**	92.0 (225.5)
**All-cause mortality (N, (%))**	4 (44.4)
**Cause of death (N, (%)) (*N*** **= 4)**	
Sepsis	3 (75.5)
No response to PLEX	1 (25.5)

Values are expressed as median (interquartile range (IQR)) or number (percentage)

*ANCA* antineutrophil cytoplasmic antibody, *AAV* antineutrophil cytoplasmic antibody-associated vasculitis, *PLEX* plasma exchange, *MPA* microscopic polyangiitis, *GPA* granulomatosis with polyangiitis, *EGPA* eosinophilic GPA; MPO: myeloperoxidase, *PR3* proteinase 3, *GBM* glomerular basement membrane, *BVAS* Birmingham vasculitis activity score, *FFS* five factor score, *ENT* ear nose throat, *DAH* diffuse alveolar haemorrhage, *RPGN* rapidly progressive glomerulonephritis, *CYC* cyclophosphamide, *RTX* rituximab, *AZA* azathioprine, *MMF* mycophenolate mofetil, *TAC* tacrolimus

### Predictors for all-cause mortality in AAV patients receiving PLEX

We assessed the predictive value of each variable for all-cause mortality using the univariable Cox hazards model analysis. MPO-ANCA exhibited a high HR for all-cause mortality; however, it was not statistically significant (HR 5.710, *P* = 0.143). In addition, neither DAH nor RPGN was associated with all-cause mortality. Among the immunosuppressive drugs administered during follow-up, RTX exhibited a tendency to reduce the rate of all-cause mortality; however, this result was not statistically significant (HR 0.209, *P* = 0.177) (Table 2).

**Table 2:** Univariable Cox hazards model analysis of variables for all-cause mortality

Variables	HR	95% CI	***P*** value
**Demographic data at PLEX**			
Age	1.007	0.952, 1.064	0.820
Male gender	1.330	0.186, 9.528	0.777
**AAV variants**	0.016	0.000, 60.511	0.327
**ANCA within 4 weeks before PLEX**			
MPO-ANCA	5.710	0.556, 58.595	0.143
PR3-ANCA	0.027	0.000, 231.881	0.434
ANCA negativity	0.676	0.069, 6.581	0.736
**AAV related indices at PLEX**			
BVAS	0.940	0.800, 1.104	0.450
FFS	1.247	0.423, 3.678	0.689
**Clinical manifestations at PLEX**			
General	N/A		
Cutaneous	0.041	0.000, 5,961,414.0	0.739
Mucous membrane/Eyes	N/A		
ENT	0.024	0.000, 119.450	0.391
Pulmonary	1.008	0.103, 9.837	0.995
Cardiovascular	0.491	0.050, 4.786	0.540
Abdominal	N/A		
Renal	0.649	0.067, 6.322	0.710
Nervous systemic	0.993	0.102, 9.69.	0.995
**Reason for PLEX**			
DAH vs. RPGN	1.544	0.139, 17.193	0.724
**Administered immunosuppressant drugs during follow-up**			
CYC	1.893	0.194, 18.490	0.583
RTX	0.209	0.022, 2.024	0.177
AZA	7.483	0.467, 119.824	0.155
MMF	0.038	0.000, 5830.207	0.592
TAC	N/A		
None	1.541	0.158, 15.021	0.710

*PLEX* plasma exchange, *ANCA* antineutrophil cytoplasmic antibody, *AAV* antineutrophil cytoplasmic antibody-associated vasculitis, *MPO* myeloperoxidase, *PR3* proteinase 3, *BVAS* Birmingham vasculitis activity score, *FFS* five factor score, *ENT* ear nose throat, *DAH* diffuse alveolar haemorrhage, *RPGN* rapidly progressive glomerulonephritis, *CYC* cyclophosphamide, *RTX* rituximab, *AZA* azathioprine, *MMF* mycophenolate mofetil, *TAC* tacrolimus

### Comparison between patients with DAH receiving PLEX and those not receiving

**Fig. 1:**
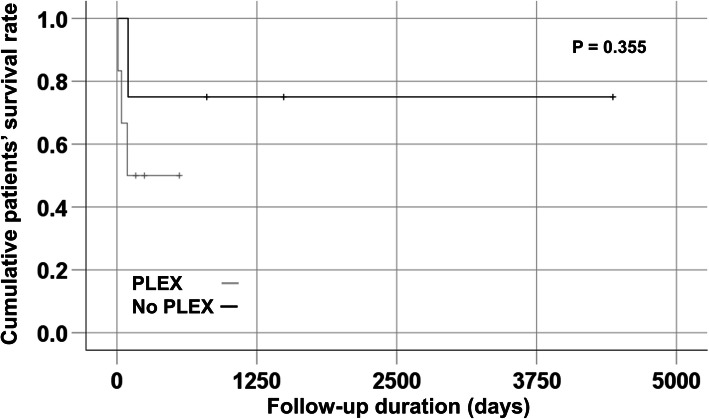
Comparison of the cumulative patients’ survival rate between patients with DAH receiving PLEX and those not receiving PLEX. No significant difference was observed between the two groups, which suggest that PLEX had no survival benefit for DAH in AAV patients. DAH: diffuse alveolar haemorrhage; PLEX: plasma exchange; AAV: antineutrophil cytoplasmic antibody-associated vasculitis

As shown in Table 3, we compared variables between patients with DAH receiving PLEX and those not receiving PLEX. There were no significant differences in demographic data, AAV variants, ANCAs, AAV-specific indices and immunosuppressive drugs administered between the two groups. In addition, the follow-up duration and the rate of all-cause mortality did not differ significantly. We also compared the cumulative patients’ survival rate between patients with DAH receiving PLEX and those not receiving PLEX using the Kaplan-Meier survival analysis to assess the survival-benefit of PLEX for DAH. However, we found no significant difference between the two groups, which suggested that PLEX had no survival benefit for DAH in AAV patients (Fig. 1).

**Table 3:** Comparison of variables between patients with DAH receiving PLEX and those not receiving

Variables	Patients with DAH not receiving PLEX (***N*** = 4)	Patients with DAH receiving PLEX (***N*** = 6)	***P*** value
**Demographic data at DAH**			
Age (years)	56.5 (21.0)	65.5 (34.0)	0.807
Male gender (N, (%))	2 (50.0)	3 (50.0)	1.000
**MPA vs. GPA (N, (%))**	3 (75.0)	4 (66.7)	0.778
**ANCA within 4 weeks before DAH (N, (%))**			
MPO-ANCA	3 (75.0)	2 (33.3)	0.197
PR3-ANCA	1 (25.0)	2 (33.3)	0.778
ANCA negativity	0 (0)	2 (33.3)	0197
**AAV related indices at DAH**			
BVAS	16.0 (17.0)	15.0 (13.5)	1.000
FFS	2.0 (0)	2.5 (2.25)	1.000
**Steroid pulse at DHA (N, (%))**	4 (100)	6 (100)	N/A
**Administered immunosuppressive drugs (N, (%))**			
CYC	4 (100)	5 (83.3)	0.389
RTX	0 (0)	2 (33.3)	0.197
AZA	3 (75.0)	1 (16.7)	0.065
MMF	0 (0)	2 (33.3)	0.197
TAC	0 (0)	1 (16.7)	0.389
None	0 (0)	1 (16.7)	0.389
**Follow-up duration (days)**	1145.5 (3421.5)	130.0 (291.8)	0.080
**All-cause mortality (N, (%))**	1 (25.0)	3 (50.0)	0.429

Values are expressed as median (interquartile range (IQR)) or number (percentage)

*DAH* diffuse alveolar haemorrhage, *PLEX* plasma exchange, *MPA* microscopic polyangiitis, *GPA* granulomatosis with polyangiitis, *ANCA* antineutrophil cytoplasmic antibody, *MPO* myeloperoxidase, *PR3* proteinase 3, *AAV* antineutrophil cytoplasmic antibody-associated vasculitis, *BVAS* Birmingham vasculitis activity score, *FFS* five factor score, *CYC* cyclophosphamide, *RTX* rituximab, *AZA* azathioprine, *MMF* mycophenolate mofetil, *TAC* tacrolimus

## Discussion

In this study, we arrived at two conclusions regarding the efficacy of PLEX on life-threatening AAV. Firstly, in terms of the rate of and predictors for all-cause mortality in AAV patients receiving PLEX, the rate of all-cause mortality was found to be 44.4%; however, no significant predictor for all-cause mortality was determined. In the MEPEX trial, the rates of all-cause mortality at 3 and 12 months were 16 and 27%, respectively, in the PLEX group, which is reflective of renal involvement as a serious manifestation in combination with a high risk of infection owing to immunosuppressive therapy [5]. Meanwhile, the PEXIVAS trial included two groups: 352 patients in the PLEX group and 352 in the no PLEX group based on glucocorticoid therapy. The rate of all-cause mortality and end-stage renal disease (ESRD) occurrence was 28.4% in the PLEX group and 31.0% in the no PLEX group. The HR of PLEX on all-cause mortality compared to that of no PLEX was 0.87 (95% confidence interval 0.58–1.29). Therefore, PLEX did not have any influence on the rate of all-cause mortality or ESRD occurrence in AAV patients [8]. It could be assumed that the very high mortality rate might interfere and offset the statistical significance of predictors of all-cause mortality after performing PLEX. In addition, this may have two clinical meanings: first, the therapeutic efficacy of PLEX might not be as high as was expected. Second, the severity of AAV might exceed the therapeutic potential of PLEX on AAV.

Secondly, we compared the survival-benefit of PLEX for DAH between patients with DAH receiving PLEX and those not receiving PLEX. Most previous studies on the efficacy of PLEX were conducted in AAV patients with kidney involvement. Two representative clinical trials, such as MEPEX and PEXIVAS, reported the conflicting efficacy of PLEX on RPGN [5, 8]. As for AAV patients with reduced kidney function due to RPGN, there are alternative treatment-modalities in addition to PLEX: transient renal replacement together with combination therapy comprising a high dose glucocorticoid and either CYC or RTX may improve RPGN [3, 4]. However, as for patients with DAH, there is no alternative treatment-modality in addition to PLEX because DAH is more rapidly progressive and fatal than RPGN. Therefore, it seems impossible to design and conduct randomised case-controlled clinical trials in AAV patients with urgent DAH, unlike that with RPGN.

There was an observational case series investigating the efficacy of PLEX on DAH in 12 AAV patients who were admitted to ICU. The authors demonstrated that PLEX together with a combination of glucocorticoid and immunosuppressive drugs might have a benefit to improve both the respiratory dysfunction and AAV-related DAH in AAV patients, although one patient died. In this study, 10 AAV patients with DAH, of whom six patients received PLEX, were retrospectively analysed [10]. However, unlike this previous study, our current study did not find any efficacy of PLEX for DAH with regard to the improvement of all-cause mortality. We considered the follow-up period as the reason for this discrepancy. The previous study evaluated the SpO2/FiO2 ratio and assessed the mechanical ventilation mode hourly for 7 days. However, they did not evaluate all-cause mortality after extubation and during follow-up [10]. Thus, it could not be easily accepted that PLEX is beneficial for the improvement in the prognosis of AAV-related DAH. In contrast, the median follow-up duration of our study was significantly longer than that of the previous study (1145.5 days for four patients not receiving PLEX and 130.0 days for six patients receiving PLEX). Of the four deceased AAV patients with DAH, three patients died of sepsis due to secondary pneumonia and one patient died because of the rapid progression of DAH owing to the ineffectiveness of PLEX (Table 3).

Considering the results of both our study and previous studies, we would suggest the use of therapeutic strategies depending on the time-course. Firstly, within 1 or 2 weeks from DAH development, PLEX along with combination comprising glucocorticoid with CYC or RTX should be promptly initiated under close observation in the intensive care unit. This strategy is expected to reduce the rate of all-cause mortality from 8.3 to 10.0%. Next, after 2 weeks from DAH development, the most common reason for death was secondary pneumonia and sepsis. Therefore, the monitoring of pneumonia occurrence, the use of preventive antibiotics and the choice of dose or type of immunosuppressive drugs should be carefully considered.

Our study has several limitations. Firstly, we provided a narrative report rather than an analytical one as the number of patients who received PLEX was too small to conduct a subgroup analysis. In particular, for this reason, we realise that the reliability of the Cox proportional analysis of this study might be limited and low. Secondly, we could not obtain sufficient information from the medical record such as PLEX technique, ventilator modes and lung-involvement pattern owing to the limitation of the retrospective study design. Thirdly, this study analysed data that had been accumulated over 8 years. During this period, the considerable improvement has taken place not only in the overall prognosis of AAV patients but also in the accuracy of ANCA tests and the efficacy of immunosuppressive drugs. Therefore, this might influence the results of this retrospective and observational study. Despite these limitations, we believe that our study, which is based on the largest cohort in Korea, could provide valuable information on PLEX in Korean patients with AAV. Therefore, this study has clinical significance as a pilot study.

## Conclusions

Nine of 212 AAV patients (4.3%) received PLEX and the rate of all-cause mortality in nine AAV patients receiving PLEX was found to be 44.4%. DAH was the most common reason for using PLEX; however, the notion that PLEX is beneficial for the improvement in the prognosis of AAV-related DAH was deemed controversial.

## Data Availability

The datasets used and/or analysed during the current study are available from the corresponding author on reasonable request.

## Abbreviations

ANCA: Antineutrophil cytoplasmic antibody
AAV: ANCA-associated vasculitis
MPA: Microscopic polyangiitis
GPA: Granulomatosis with polyangiitis
EGPA: Eosinophilic GPA
EULAR: European League Against Rheumatism
ERA-EDTA: European Renal Association-European Dialysis and Transplant Association
CYC: Cyclophosphamide
RTX: Rituximab
RPGN: Rapid progressive glomerulonephritis
DAH: Diffuse alveolar haemorrhage
PLEX: Plasma exchange
KDIGO: Kidney Disease: Improving Global Outcomes
GBM: Glomerular basement membrane
MEPEX: Methylprednisolone versus Plasma Exchange
PEXIVAS: Plasma Exchange and Glucocorticoids for Treatment of AAV
BVAS: Birmingham vasculitis activity score
FFS: Five factor score
MPO: Myeloperoxidase
PR3: Proteinase 3
IQR: Interquartile range
HR: Hazard ratio
